# Flow synthesis and multidimensional parameter screening enables exploration and optimization of copper oxide nanoparticle synthesis†

**DOI:** 10.1039/d4na00839a

**Published:** 2024-12-03

**Authors:** Neal Munyebvu, Zarina Akhmetbayeva, Steven Dunn, Philip D. Howes

**Affiliations:** a School of Engineering, London South Bank University London SE1 0AA UK; b School of Engineering and Informatics, University of Sussex Brighton BN1 9RH UK p.d.howes@sussex.ac.uk

## Abstract

Copper-based nanoparticles (NPs) are highly valued for their wide-ranging applications, with particular significance in CO_2_ reduction. However current synthesis methods encounter challenges in scalability, batch-to-batch variation, and high energy costs. In this work, we describe a novel continuous flow synthesis approach performed at room temperature to help address these issues, producing spherical, colloidally stable copper(ii) oxide (CuO) NPs. This approach leverages stabilizing ligands like oleic acid, oleylamine, and soy-lecithin, a novel choice for CuO NPs. The automated flow platform facilitates facile, real-time parameter screening of Cu-based nanomaterials using optical spectroscopy, achieving rapid optimization of NP properties including size, size dispersity, and colloidal stability through tuning of reaction parameters. This study highlights the potential of continuous flow synthesis for efficient parameter exploration to accelerate understanding, optimization, and eventually enable scale-up of copper-based NPs. This promises significant benefits for various sectors, including energy, healthcare, and environmental conservation, by enabling reliable production with reduced energy and cost requirements.

## Abstract 2

Copper-based nanoparticles (NPs) are highly valued for their wide-ranging applications, with particular significance in CO_2_ reduction. This paper explores a multiparametric approach to CuO synthesis using a flow reactor.

## Graphical abstract

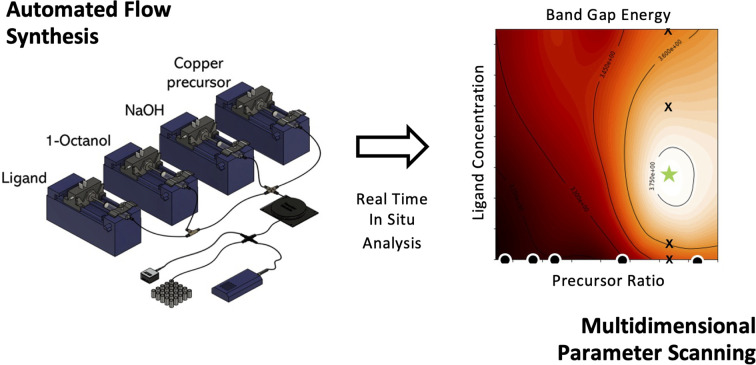

## 1 Introduction

Copper (Cu)-based nanoparticles (NPs) are receiving increasing interest for various applications, with particular significance in CO_2_ reduction,^1^ antimicrobials,^2^ and cancer therapy.^3^ They exhibit a range of useful physical and chemical properties, while benefiting from an abundance of natural feedstock, and exhibiting low toxicity to humans. These particles are low in cost to produce, with a wide variety of different possible precursors and synthetic routes.^4^

Copper(ii) oxide (CuO) is a subset of Cu-based materials that has received significant attention in the literature, due to its behavior as a p-type semiconductor. The ability to tune the size of CuO in the nanoscale has been shown to enable the engineering of its band gap energy,^5^ making the NPs an interesting material for use in electronic and optoelectronic devices. For example, Siddiqui *et al.* used CuO NPs to enhance the behavior of organic solar cells.^6^ The addition of CuO helped adjust the morphology of the active layer, leading to an increase in device efficiency. The study also noted a shift in the absorption spectrum to the visible region, improving light absorption.

Several CuO NP syntheses have been established, ranging from top-down (milling, grinding.) to bottom-up approaches (wet-chemical, electrochemical, sonochemical, *etc.*).^4^ From these, wet-chemical methods are particularly desirable, allowing precise control of NP shape and size, solution processibility of resultant products, and general low cost. Here, the copper ion complex is generally reduced *via* a reducing agent (*e.g.* hydrazides, hydroxides), or used as a precursor in a precipitation reaction within aqueous or organic solvents.

For nanomaterials, it is generally preferable for products to be uniform in size, shape, and size distribution, as well as stabilized against aggregation and sedimentation. Typical syntheses of CuO NPs employ organic stabilizing molecules to facilitate precursor dissolution, modulate particle nucleation and growth, and then enhance colloidal stability. Ligand engineering provides an excellent means of achieving size and shape control. For example, acetic acid has been used to maintain the spherical shape of CuO NPs at elevated temperatures,^7^ while more complex molecules such as hexadecyltrimethylammonium bromide have been reported to improve the colloidal stability of CuO.^8^

To date, there has been very little investigation and exploration of different ligands and their effects on CuO NP synthesis and product properties, which thus appears to be an area of great potential. Oleic acid (OAc) and oleylamine (OAm) are stable long-chain ligand molecules that are particularly effective when paired, as they each provide different surface binding modes while possessing very similar chain lengths. Their dynamic binding nature means they are extremely versatile and have been used for a plethora of nanomaterials.^9^ In contrast to OAc/OAm, lecithin is a zwitterionic ligand that provides dual binding modes with a long chain backbone and achieves strong binding capable of stabilizing challenging semiconductor NPs with ionic behavior (*e.g.* lead halide perovskites).^10,11^ The contrasting natures of these ligand systems provide an interesting comparison for study in pursuit of a versatile and controlled synthesis of CuO NPs.

Current syntheses of Cu-based nanomaterials rely overwhelmingly on traditional batch synthesis. However, the inherent low throughput of this approach limits exploration of the reaction parameter space, which is extensive given the wide range of different precursors, reducing agents, ligands, temperatures, and reaction processes and times. This severely limits product innovation and optimization. While significant research has been conducted in parallelization and the use of robotic systems to achieve high throughput optimization in batch, flow chemistry has also emerged as an attractive option to overcome this challenge.^12^ Although flow synthesis of metal and metal oxide nanoparticles is well studied in general,^13–16^ there has been relatively little progress with Cu-based NPs. Although Cu-based NPs have been synthesized under continuous flow conditions,^17^ specific focus on CuO has been limited to approaches requiring the use of millifluidic channels and using specific and high energy activation techniques *e.g.*, microwave-assisted^18^ or laser ablation synthesis.^19^

Combining the concepts of flow chemistry, microfluidic components and in-line analytics opens avenues to rapid and efficient reaction optimization.^20–23^ Here, instrumentation such as precision syringe pumps and in-line heaters allows dynamic control of a wide range of reaction parameters, while in-line analytics (such as optical spectroscopy^24^) yields real time data on reaction product properties.^25,26^ This data can be used to map a multidimensional parameter space, or it can be processed in real time for algorithm-guided product optimization.^27^ while hardware and software development in this space is well-established and continues apace,^28^ there are still few examples of such systems being used in nanochemistry research beyond proof-of-principle.

Importantly, it must be acknowledged that flow is not inherently superior to batch approaches and is not suitable in all cases. It is a tool that has helped supplement more traditional processes for the benefits highlighted above. However, drawbacks do exist that mean that flow reactors can require significant optimization for the best quality product.

In this work, we demonstrate a facile, room temperature continuous flow synthesis of copper(ii) oxide (CuO) NPs. We report, for the first time, spherical CuO NPs synthesized under flow conditions, and directly compare them to an analogous batch synthesis. We then develop this further and introduce stabilizing ligands under continuous flow to understand changes in the size and colloidal stability profile of synthesized particles using optical measurements. To the best of our knowledge, this is the first demonstration of the use of soy lecithin for the synthesis and stabilization of CuO NPs, and represents the first time rapid parameter screening has been used to optimize CuO NP synthesis, identifying the ligand and NaOH concentrations that produce NPs with minimal size and polydispersity index (PDI). Beyond CuO NPs, the developed approach is versatile and accessible, and holds significance for a wide range of colloidal metal and metal oxide nanoparticle syntheses.

## 2 Results and discussion

### 2.1 Residence time determination

Initial work focused on novel development of a suitable flow reaction for CuO NPs. Based on a previously reported synthesis of spherical CuO NPs performed at elevated temperature,^7^ we sought to develop an understanding of the appropriate time frame for the synthesis to be adapted to a flow platform for fast parameter screening at room temperature.^29^ The ability to control the time frame of a reaction within a flow synthesis is critical, and a balance needs to be struck. Faster reactions are more time efficient and easier to deal with in a flow system, however it is common to encounter difficulties controlling NP product quality *e.g.* wide size distributions. Slower reactions are often easier to control, but extended residence times (beyond 15–20 min per experiment) are harder to accommodate in a flow system, can result in problematic reactor fouling,^30^ and reduce utility of rapid parameter screening in product optimization.

Room temperature reactions to CuO NPs have been reported previously, but at extended timescales,.^31–36^ Adapting these to flow requires a deeper understanding of the reactions, and ideally ways on controlling them. Previous literature indicates that increasing the concentration of the NaOH relative to the copper salt precursor decreases the reaction time.^8^ Accordingly, using 1-octanol as the primary solvent, we scanned five different molar ratios (copper acetate to sodium hydroxide) using an *in situ* UV-visible (UV-vis) absorption spectroscopy setup, recording spectra at 1 s intervals for 5 min to provide an overview of reaction dynamics (Fig. S1†).

**Fig. 1:**
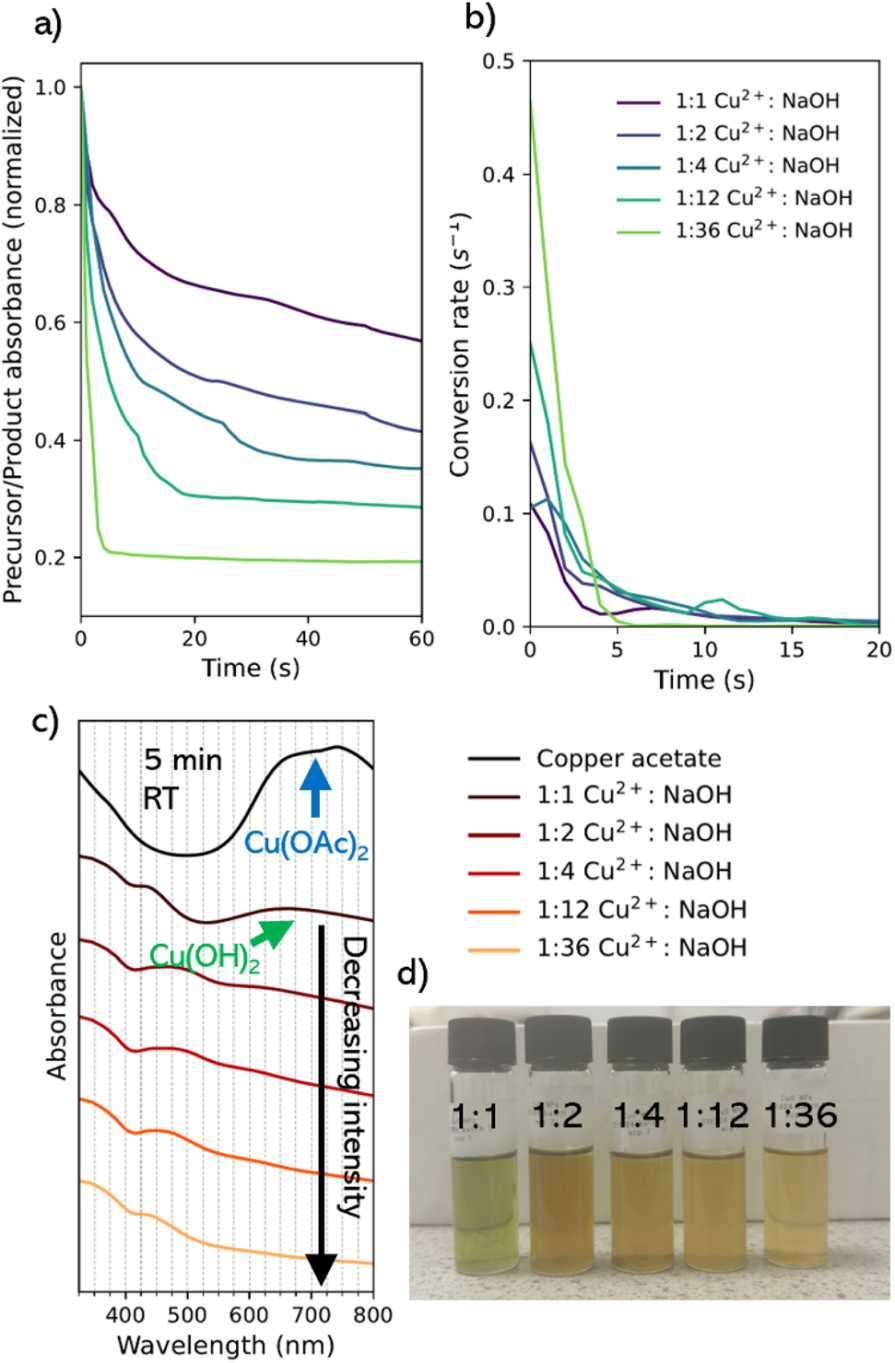
(a)The ratio of the intensity of product peak against the precursor peak over the first 60 seconds of injection of NaOH. A Savitsky–Golay filter was applied to smooth the data and clearly interpret the rate of change. (b) Conversion rate of the reaction calculated from smoothed time course data. (c) Final UV-vis absorption spectrum for the colloidal solutions after 5 min reaction time (RT) upon the addition of various molar ratios of NaOH. (d) Photograph of the as-prepared colloidal solutions after 5 min reaction (pre-purification) under batch conditions.

The time taken for the precursor to be consumed in the reaction was directly related to the ratio of NaOH added while the Cu^2+^ concentration was kept constant. The literature indicates the characteristic absorbance peak for CuO NPs is in the 280–320 nm range. Our *in situ* spectrophotometer was capable of measuring between 325–800 nm, so we compared the absorbance of the Cu(OAc)_2_ precursor peak position at 697 nm against the position closest to the expected peak of CuO at 325 nm. With the typical spectra being a curve around this region for CuO NPs, we can expect that the relative ratio of the precursor absorbance peak against this point should decrease as the precursor (copper acetate) and intermediate (copper hydroxide) are consumed. As such, we could track the progress of the reaction and show that varying NaOH ratio against a fixed concentration of Cu^2+^ has a significant influence on the rate of reaction. A Savitsky–Golay filter was applied to smooth the data and clearly interpret the rate of change (Fig. S2†). Here, the lower the value, the greater the relative absorbance of CuO within the solution, relative to Cu(OAc)_2_ (Fig. 1a). This would correspond to the concentration of CuO NP within the solution relative to the concentration of the precursor solution as described by the Beer–Lambert law.^37^ For a 1 : 1 Cu^2+^ : NaOH ratio, there is a transition from the copper acetate to the expected intermediate state (copper hydroxide, Cu(OH)_2_) at a rate of 0.1089 s^−1^ (Fig. 1b). This was seen by a shift in the peak position to a broad peak in the visible region near 660 nm (Fig. 1c) assigned to the Cu(ii) d–d transition in copper hydroxide.^38^ After 5 min the starting material does not experience full conversion to the final CuO product and the presence of the Cu(OH)_2_ peak remains, alongside the blue-green color of the reaction mixture (Fig. 1d).^39^

As the molar ratio of the NaOH to Cu^2+^ is increased from 1 to 36, the conversion rate from the starting material to the final absorption spectrum significantly increased (Fig. 1b). The 1 : 36 condition transition occurs rapidly at a rate of 0.4641 s^−1^, alongside an immediate transition from blue to a dark brown solution with the complete transition of the copper precursor within 5 min (Fig. 1d). This was explained as result of the rate of reaction. At low concentrations of NaOH, there is a lower probability for interaction with the Cu^2+^ precursor, resulting in a high concentration of larger-sized intermediate particles, as a result of slow nucleation. Upon transition to the CuO species over time, particles appear as large dark brown metallic clusters. At high concentrations, where the NaOH is in excess, a high rate of reaction means that nucleation, growth, and oxidation of the NPs occur almost instantaneously. This corresponds to data in existing syntheses studying the variation of NaOH concentration against the Cu^2+^ precursor.^33,40^

To confirm the presence of CuO (after purification and drying) the XRD pattern of the 1 : 12 product was obtained (Fig. S5†), with the data in good agreement with Crystallography Open Database diffraction data for crystalline monoclinic CuO (COD 9016057). EDS analysis of the batch sample (Fig. S7†) also showed the presence of CuO with an approximate ratio of 1 : 1 between copper and oxygen atoms.

From this, we could confirm that a synthesis of CuO NPs at room temperature, transferable to continuous flow, could be achieved with a 5–10 min timescale. Alongside this, we could achieve a controllable reaction time *via* tuning the molar ratio of NaOH at fixed Cu^2+^ concentration, giving suitable conditions for rapid parameter screening under flow conditions.

### 2.2 Comparison of CuO synthesis in batch and flow

**Fig. 2:**
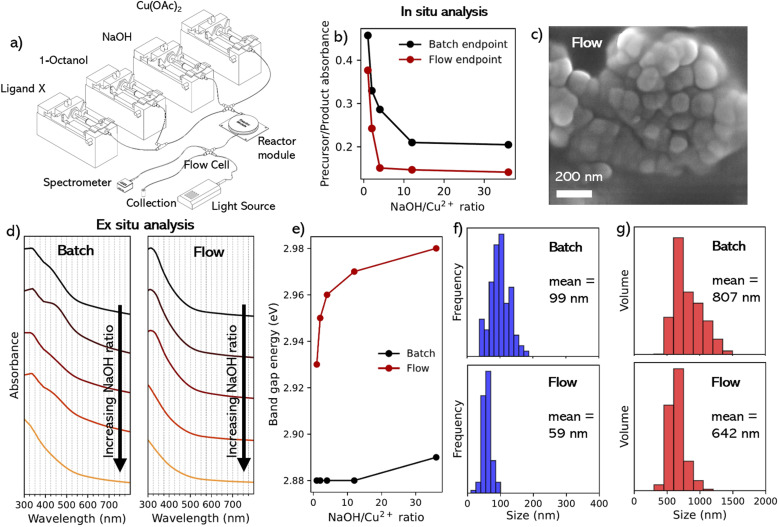
(a) Schematic of the flow reactor used for the synthesis of CuO NPs during this study. (b) Plot of the ratio of the intensity of product peak against the precursor peak for the UV-vis taken after 5 minutes for both batch and flow conditions. (c) SEM image for the CuO NPs synthesized using a 1 : 12 Cu^2+^ : NaOH ratio under flow conditions at 87 K × magnification. (d) UV-vis absorption spectrum for the colloidal solutions made under both batch and conditions post-purification. (e) Band gap energies of the colloidal solutions made under both batch and flow conditions post-purification. (f) Particle size histograms (blue) calculated from SEM image analysis for CuO NPs synthesized using 1 : 12 Cu^2+^ : NaOH ratio under both batch and flow conditions. (g) Particle size histograms (red) calculated using DLS for CuO NPs synthesized using 1 : 12 Cu^2+^ : NaOH ratio under both batch and flow conditions.

Work then focused on adapting the batch synthesis to a continuous flow platform. An illustration of the flow reactor used in the current work is shown in Fig. 2a. This setup allowed precise and individual control over the precursor and ligand concentrations (and therefore ratios). These can be changed in real time, enabling reaction parameter scanning and mapping.

NaOH/Cu^2+^ ratios of 1, 2, 4, 12, and 36 were used to synthesize CuO NPs under continuous flow conditions to evaluate the impact of flow conditions on the endpoint of a 5 min reaction. Synthesis of NPs under flow conditions was confirmed *via* SEM (Fig. 2c). EDS analysis (of the 1 : 12 flow sample, Fig. S13†) again confirmed the presence of CuO, with a 1 : 1 atomic ratio between copper and oxygen, which closely matched the batch sample in the same conditions (Fig. S7†).

As mentioned, in the batch reaction at 1 : 1 Cu^2+^ : NaOH, *in situ* absorption analysis revealed the presence of a peak corresponding to the reaction intermediate Cu(OH)_2_ (Fig. 1c). UV-vis absorption spectra were also recorded *in situ* after 5 min of continuous flow (Fig. S9b†), which allowed comparison with the *in situ* batch experiments (Fig. S9a†). This could be seen in Fig. 2b where, similar to the residence time determination experiments, we compared the absorbance of the precursor peak position (697 nm) against the nearest measurable position in the UV region corresponding to the CuO peak (325 nm). However, we only looked at the end-point of both regimes to understand the relative absorbance of precursor species against the final CuO species after 5 min. We can see for all NaOH/Cu^2+^ ratios, the flow reactions showed more complete conversion in comparison to the batch synthesis within the same reaction time. In addition, after 5 min a plateau of relative absorbance was seen at a lower NaOH/Cu^2+^ ratio under flow conditions (4) compared to batch conditions (12).

UV-vis absorption spectra were also recorded *ex situ* subsequent to purification and redispersion in 1-octanol, for both batch and flow samples (Fig. 2d). As can be seen from these spectra, there was less pronounced shoulder peaks between 400–500 nm in the flow samples, which gave smooth absorption spectra. This shoulder peak has been previously attributed to surface plasmon resonance from copper atoms on the surface of CuO.^8^ From the *ex situ* UV-analysis, the direct band gap energies were estimated (eqn (S2)†) from their corresponding Tauc plots (Fig. S10†), and plotted in Fig. 2e. From the batch reaction, there was little change in band gap energy for the different samples (remaining at 2.88–2.89 eV), regardless of the Cu^2+^ : NaOH ratio. This is consistent with an existing literature value of 2.85 eV for CuO NPs synthesized under similar conditions.^41^ In contrast, the flow conditions exhibited higher band gap values and a greater variation through the different reaction conditions, relative to the batch experiments (2.93–2.98 eV). This was an initial indication that the flow reaction may yield smaller NPs relative to the batch reaction, as it has been reported that larger direct band gap energies correspond to smaller sizes for CuO NPs.^5,40,42^ This was attributed to enhanced mass transport and increased rate of diffusion afforded by the higher surface area-to-volume ratio under microfluidic conditions, resulting in faster, more uniform nucleation, and yielding smaller and less polydisperse NPs, as seen for both metal oxide and quantum dot NPs.^43,44^

NP size was investigated by XRD and SEM using the 1 : 12 (Cu^2+^ : NaOH) samples under batch and flow conditions. For the batch synthesis, the XRD average grain size was calculated as 16.18 nm, according to the Debye–Scherrer equation (eqn (S1)†), with the SEM diameter (Fig. S6†) yielding an average of 99 nm (2f) with a standard deviation of 29.3 nm. However, significant aggregation in the batch samples made diameter estimation from SEM difficult. For the flow synthesis, analysis of the powder obtained under XRD showed strong peaks corresponding to the (002) and (111) lattice planes, the primary peaks typical for the presence of CuO NPs (Fig. S11†), with peak broadening indicating a decrease in the crystal size. This yielded a calculated average grain size of 4.49 nm, smaller than that measured for batch conditions. This also corresponds to the shift in size measured in the SEM with a smaller mean particle size of the flow sample of 59 nm, which also showed a narrower size distribution with a standard deviation of 10.6 nm (Fig. 2f) This significant difference in size between the XRD and SEM indicates a highly polycrystalline structure of the flow-synthesized NPs.

From the SEM images, it was clear that CuO NPs were approximately spherical when synthesized under both regimes. This is not typical for syntheses using NaOH to react with Cu^2+^ in the absence of other additives or at room temperature.^7,31–36^ In contrast to previous studies, this morphology may be the result of several factors, including the smaller relative reaction volumes, pH effects, solvent effects and the reduced reaction/aging time of the NP products. We hypothesize it is a combination of the proposed factors, with solvent effects having the most significant impact due to differences in dielectric permittivities that reduce the electrostatic interactions between charged species in 1-octanol (dielectric permittivity = 9.86)^45^ compared to typical syntheses described above that use water or ethanol (dielectric permittivity 79.99 and 25.02, respectively).^46^ This was seen in a previous study from Ganga *et al.* that looked at the solvent effect on CuO NP morphology during synthesis; switching from distilled water (DI) to DI : ethanol mixture changed morphology of CuO from nanorods to near-spherical particles.^40^ However, that experiment was carried out at elevated temperatures (70 °C) compared to the room-temperature approach explored in this study.

Despite the reduced size and improved uniformity of NPs synthesized under flow conditions, there was still a significant degree of aggregation. For example, DLS analysis of the 1 : 12 Cu^2+^ : NaOH sample showed a volume mean size of 642 nm (Fig. 2g), more than an order of magnitude greater than the mean core diameter calculated from SEM analysis (59 nm), as a result of the aggregation within the NP solution. Visual observation of the products found that for the as-prepared CuO NPs, dispersions exhibited clear signs of aggregation within the organic media.

**Fig. 3:**
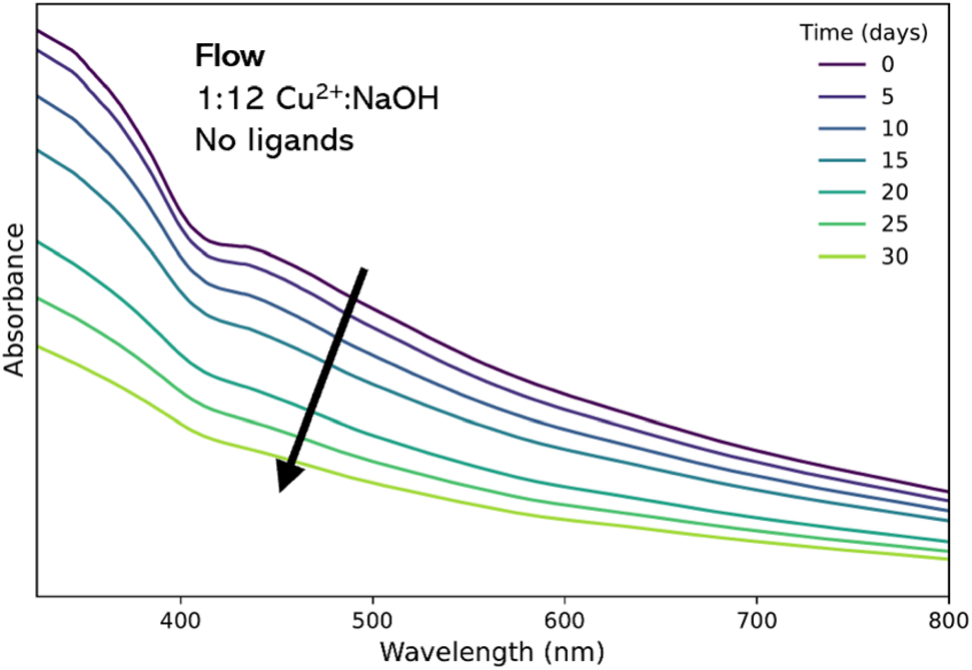
Evolution of UV-vis absorption spectra of the 1 : 12 colloidal suspension synthesized in flow recorded over 30 days.

To quantify the aggregation behavior of samples after synthesis and cleaning, *ex situ* UV-vis was used to measure the absorption spectra every 5 days for 30 days. The 1 : 12 Cu^2+^ : NaOH flow product was chosen for study. Over time, absorbance steadily decreased by approximately 50%, indicating the general instability of the material without the presence of active stabilizing strategies (Fig. 3).

The results of this part of the study indicate that when comparing the relatively simple platforms used in this work there were benefits to the flow synthesis over the batch approach, with products yielding smaller particles with narrower size distribution for the same synthesis. However, aggregation and colloidal instability remained an issue in both cases. Therefore, we moved on to investigate novel ligand combinations for CuO as a means to improve colloidal stability, as well as investigating their ability to modify the characteristics of the NPs.

### 2.3 Ligand study for flow synthesized CuO NPs

#### 2.3.1 OAc/OAm *versus* soy lecithin as capping ligands

**Fig. 4:**
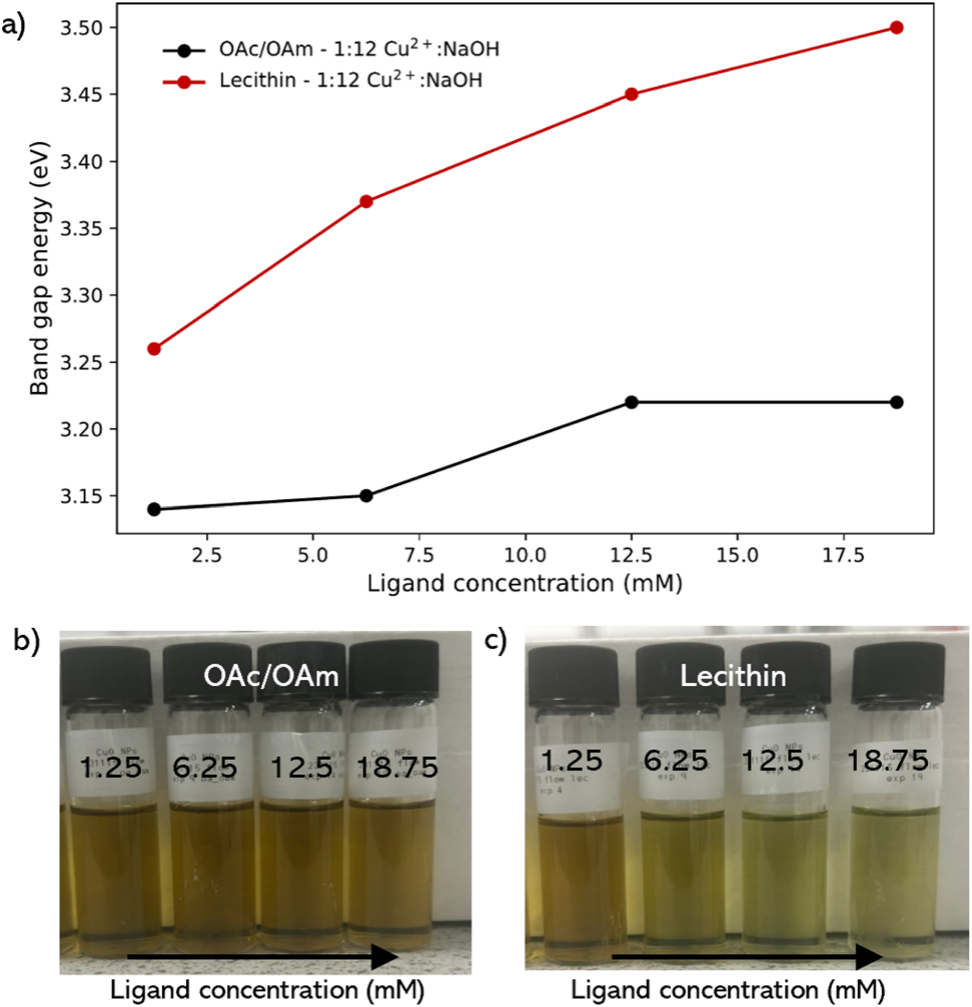
(a) corresponding band gap energies of the CuO NP colloidal solutions synthesized using OAc/OAm binary and soy-lecithin flow conditions. Photographs of CuO NPs synthesized in flow at 1 : 12 Cu^2+^ : NaOH ratio using (b) oleic acid (OAc) and oleylamine (OAm) binary mixture, and (c) soy-lecithin zwitterion at various molar concentrations.

The oleic acid and oleylamine (OAc/OAm) binary ligand pair was selected for study as it has been previously seen to act as an effective ligand strategy for various colloidal NP classes,^9^ including Cu NPs.^47^ To the best of our knowledge, this is the first time this combination has been used in the synthesis of CuO NPs. With the presence of 1.25 mM of the OAc/OAm in the reaction solution, the overall band gap energy of the NP product increased by 170 meV (Fig. 4a) relative to the CuO NPs with no ligand (Fig. 2e), indicating a decrease in the size or improvement in the dispersity of the synthesized nanoparticles. Upon increasing concentration up to an excess level (18.75 mM) of OAc/OAm relative to Cu^2+^, there was a slight increase in band gap energy of 80 meV between 1.25 mM to 18.75 mM, indicating a shift in the electronic properties of the NP solution as we increase the ligand concentration (Fig. 4b).

Lecithin was also investigated as a ligand because it has emerged as an effective ligand for colloidal nanoparticle synthesis and capping.^10^ It exhibits a zwitterionic nature over a wide pH range, containing both positive and negative charges due to the presence of a choline molecule (–(CH_2_)_2_–N^+^–(CH_3_)_3_) and a phosphate group (PO_4_^3−^) in its structure, respectively. Lecithin has been shown to achieve stronger binding compared to the binary ligand pair of OAc/OAm, and confers colloidal stability in different organic solvents.^10,11,48^ Similar to the binary pair, lecithin also showed an increase in band gap energy in contrast to CuO NPs synthesized under the conditions without additives. However, the shift was significantly greater than that of the OAc/OAm pair, with an increase of 290 meV upon introduction with 1.25 mM lecithin, greater than the total increase in band gap energy across the entire concentration range of the OAc/OAm binary ligand pair (Fig. 4a). With a steady increase of 240 meV between 1.25 mM up until an excess of lecithin was introduced, giving a total shift of above 500 meV starting from no ligand across this concentration range.

Interestingly, at 12.5 mM and 18.75 mM, corresponding to the equimolar and slight excess ratios of ligand to Cu^2+^ (12.5 mM), the colloidal solutions did not fully transition to the brown/yellow solution and instead appeared to transition to a lighter yellow/green solution (Fig. 4c). This suggests that at excess concentrations of lecithin, the ligand suppresses the reaction of the copper precursor species with NaOH, resulting in a larger concentration of precursor/intermediate species under the same conditions within the solution, indicating that the concentration of lecithin used in the synthesis needs to be carefully optimized.

The comparative analysis of OAc/OAm and soy lecithin as capping ligands for CuO nanoparticles reveals that lecithin is more effective at tuning the electronic properties of the nanoparticles. While both ligands increase the band gap energy, indicating smaller or more monodispersed nanoparticles, the effect is notably more pronounced with lecithin, which achieves a significant band gap shift of over 500 meV.

Evidently, there will be a dynamic interplay between the lecithin, the Cu(OAc)_2_ precursor, the reaction intermediate Cu(OH)_2_, the CuO nuclei and growing particles. We propose that for already small and stable NPs (*e.g.* NPs synthesized at higher NaOH/Cu^2+^ ratios), at a certain point, the further increase in ligand concentration does not necessarily result in smaller, more monodisperse nanoparticles. Upon full surface coverage of ligands on the surface of the NPs, there could no longer be further growth, and size would reach an equilibrium.^49^ The increased concentration, in this case, will instead impact the interaction between the precursor and intermediary species within the reaction solution. This may be *via* steric hindrance within solution between precursor species, or through binding of the ligand to the precursor salt ions.^50^ For strongly binding ligands, where dissociation of the ligand is less labile, this may result in a regime where both scenarios are present. Under these conditions, nucleation is both delayed (due to coordination to the precursors) and where NPs can nucleate, growth and aggregation are inhibited, resulting in a mixed NP environment with a higher PDI. This behavior has also been seen in palladium nanoparticles generated from palladium acetate.^51^ For more dynamic binding ligands, such as the OAc/OAm binary pair, labile association and disassociation of the ligands means that even when they are in excess, this equilibrium allows for nucleation and growth to proceed, although at a slower rate.^49^

From this, we concluded that the interesting behavior of the soy-lecithin ligand warranted further investigation. However, it was clear the ligand concentration would require careful optimization, and as a result, soy-lecithin was taken forward for further investigation.

### 2.4 Optimization of the lecithin-capped CuO NPs *via* rapid parameter screening

**Fig. 5:**
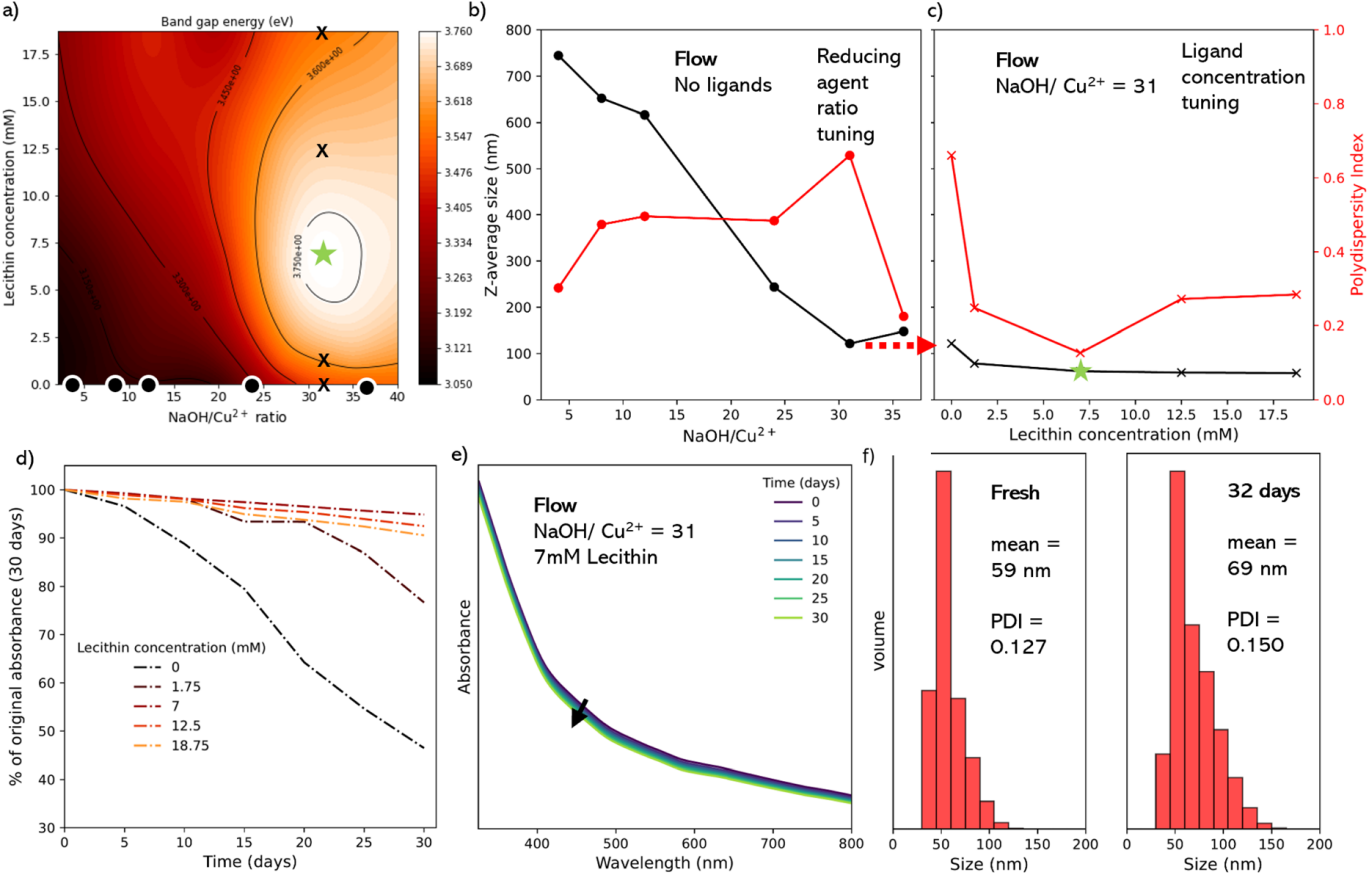
(a) Contour plot where crosses indicate positions analyzed offline in Section c. (b) Particle size (*Z*-average) of the CuO NPs synthesized at various Cu^2+^ : NaOH ratios. (c) Particle size (*Z*-average) of the CuO NPs synthesized using 1 : 24 Cu^2+^ : NaOH ratio at various lecithin concentrations. (d) Evolution of UV-vis absorption spectra of the 1 : 31 colloidal suspension synthesized in flow at various lecithin concentrations recorded over 30 days. (e) Evolution of UV-vis absorption spectra of the 7 mM lecithin CuO suspension synthesized in flow recorded over 30 days. (f) Particle size histograms calculated from DLS of the 7 mM lecithin CuO suspension – fresh and after 32 days.

A key advantage of the flow reactor is the ability to rapidly scan many conditions in a multidimensional parameter space, and map outputs as a function of inputs. This helps to understand the effect of each parameter and select optimal reaction conditions toward to a desired goal. It is well reported that key parameters such as NP band gap and size distribution are important for applications in applications such as photovoltaics and catalysis. Therefore We decided to target CuO NPs of a minimal size and PDI. We sought to control two input variables, the lecithin concentration (5 points) and NaOH concentration (5 points, represented as Cu^2+^ : NaOH molar ratios) to study their impact on the band gap energy of the CuO NPs as our output variable (as a proxy for minimizing the NP size). This gave an array of 25 data points from the equivalent number of experiments, which were then fitted using a thin plate spine, yielding the contour plot shown in Fig. 5a. We hypothesize that the peak in this response surface should correspond to the CuO NPs of the lowest diameter and PDI.

From the contour plot (in Fig. 5a), we observed a clear peak in the band gap energy (denoted by a star), corresponding to a 1 : 31 ratio of Cu^2+^ : NaOH at a lecithin concentration of 7 mM. As previous results above suggested that this peak should correspond to the minimum particle size and polydispersity index, and we sought to prove this by conducting DLS analysis on specific samples (denoted by dots and crossed in the contour plot).

First, we performed 6 experiments at the 0 mM lecithin condition (dots in Fig. 5a), corresponding to ratios of 4, 8, 12, 24, and 36, plus 31 for its correspondence with the peak (star). These samples were filtered, purified, redispersed in 1-octanol. DLS analysis confirmed that the increase in the band gap energy corresponds to a reduction in the average measured diameter of the NPs, with a decrease in the *Z*-average particle diameter of 744.5 nm at a 1 : 4 ratio, down to 108.1 nm at a 1 : 36 ratio (Fig. 5b). Further, all of these samples had a relatively high polydispersity index (PDI) between 0.225–0.661 (5b, in red) suggesting a high degree of aggregation and sedimentation, which would be expected without ligands.^52^ For highly monodisperse samples typically PDI is ≤0.1. In this work, we did expect a certain degree of polydispersity since we were exploring the trend in the behaviour of the PDI against the specific conditions used in the parametric scan. For the best possible NPs further optimization would be required.

Subsequently, we performed an additional 4 experiments with lecithin concentrations of 1.25, 7, 12.5, and 18.75 mM at 1 : 31 Cu^2+^ : NaOH ratio (crosses in Fig. 5a). Here the 7 mM sample corresponds to the contour plot peak (star). Data from these samples (Fig. 5c) showed that increasing lecithin concentration led to a reduction in the *Z*-average particle size, with the sharpest step seen between 0 mM lecithin (121.8 nm) and 1.25 mM lecithin (78.6 nm). The star condition yielded a *Z*-average diameter of 61.8 nm, with no significant difference seen with further increases in lecithin. A more dramatic effect was seen in the PDI, which dropped sharply between the 0 mM lecithin (0.661) and 1.25 mM (0.248) conditions, and reached a minimum at 7 mM lecithin (0.127). The subsequent increase in PDI with further increases in the lecithin, which corresponds well with the band energies in the contour plot, where the peak band gap energy corresponds with the minimum in the PDI, and near minimum in the particle diameter. This indicates that the overall band gap energy of the CuO NPs is dependent on both the size and polydispersity of the colloidal suspension. As such, even where the average size was smaller or comparable to the highlighted peak conditions, the presence of larger particles, aggregates, and impurities caused a decrease in the overall band gap energy of the colloidal suspension. Previous work looking at the effect of polydispersity on the band gap energy of semiconductor NPs also highlights this effect.

To evidence binding of the lecithin to the final CuO samples, we studied the purified CuO NP powders using FTIR spectroscopy (Fig. S16†), which showed characteristic behaviour of CuO for all samples studied (0, 6.25 and 18.75 mM lecithin).^53^ Upon increasing lecithin concentration, we observed a strong peak at approximately 1034 cm^−1^, which is strongly present in soy-lecithin precursor sample and has been assigned to the highly overlapped PO_2_ and P–O–C infrared active vibrations in the phosphate group.^54^

We show that it was possible to achieve rapid optimization of the CuO NP synthesis toward minimizing NP size and size dispersity by seeking to maximize the band gap energy in a multidimensional parameter scan, and that the flow reactor with inline absorption measurement was an effective means of achieving this.

### 2.5 Effect of lecithin on colloidal stability of CuO nanoparticles

Finally, we sought to assess the colloidal stability of the lecithin-capped CuO NPs, looking toward their application as a colloidal ink for application device fabrication. To determine the aggregation behavior of the colloidal solutions directly, UV-vis evolution analysis was again used as a proxy for particle sedimentation (Fig. 5d), for several concentrations of lecithin (0, 1.75, 7, 12.5 and 18.75 mM). This was done by repeating the UV-vis absorption spectra every 5 days for 30 days. As seen in Fig. 5d, the best performing sample was the 7 mM (corresponding to the green star sample), whose absorption (at 325 nm) only 5.2% in 30 days, compared to 53.5% and 23.3% for no ligand (Fig. 3) and 1.25 mM lecithin samples (Fig. S17†), respectively. The 12.5 and 18.75 mM samples showed an increase in stability relative to very low lecithin samples, however not as stable as the 7 mM sample with a 7.5% and 9.5% decrease in maximum absorbance, respectively (Fig. S17†).

After 32 days, DLS was used to again assess the optimized 7 mM product, and it could be seen that the volume mean of the sample increased by just 10 nm after a month, with a slight increase in the PDI from 0.127 to 0.150 (Fig. 5f), indicating the presence of stable CuO particles and confirming the effect of lecithin as a viable and effective ligand for CuO NPs.

## 3 Conclusions

This study has demonstrated a novel room temperature, facile, and reproducible method for synthesizing copper(ii) oxide (CuO) nanoparticles (NPs) using a continuous flow reactor. Compared to the analogous batch synthesis, the developed flow synthesis method yielded smaller CuO nanoparticles (59 nm compared to 99 nm) with narrower size dispersity, as observed under SEM. Additionally, the developed flow synthesis eliminates the need for high temperatures, pressures, or complex mixing techniques, making it an environmentally friendly and scalable option for producing high-quality CuO nanoparticles.

This work successfully integrated the use of ligands such as the oleic acid/oleylamine (OAc/OAm) pair and soy lecithin in the synthesis process. The novel introduction of lecithin not only facilitated control over the electronic properties of the nanoparticles but also significantly improved the colloidal stability, extending the potential applications of these nanoparticles. A mechanism for the ligand interaction with the CuO NPs during nucleation and growth was also proposed based on the literature, and this hypothesis will be explored and validated in a focused study on the ligand chemistry of the synthesized CuO NPs.

The utilization of a flow reactor with microfluidic components enabled rapid parameter scanning in a multidimensional parameter space. This capability allowed for the optimization of reaction conditions, specifically identifying the ligand and NaOH concentrations that produce CuO NPs with minimal size and polydispersity index (PDI). The optimized conditions achieved in this study resulted in nanoparticles with an average diameter of *ca.* 60 nm and a low PDI of 0.127 indicating a narrow dispersity of NPs which remained stable for 32 days.

We also note that reactor fouling is an important consideration in the development of flow reactors and reaction for the synthesis of nanomaterials^30^ Whilst fouling and its effects were not observed during the current work, we were judicious in flushing the reactor between experiments and periodically replacing tubing, and experiments were run typically less than 2 h. We advise that such precautions are important in developing and running flow synthesis of nanomaterials, and point to the fact that there are various approaches to avoid fouling.^55^

The findings from this study underscore the significant advantages of using continuous flow chemistry for nanoparticle synthesis, particularly in terms of efficiency and control over the synthesis process. The results are not only significant for CuO nanoparticles, as the approach could be extended to a wider range of metal and metal oxide nanoparticles. The methodology we have outlined represents and accessible and effective approach to synthesis optimization for colloidal nanoparticles, with the ability to rapidly modify reaction conditions and immediately observe the effects on nanoparticle properties, providing a powerful tool reaction and product development.

## 4 Materials and methods

### 4.1 Materials

Copper(ii) acetate monohydrate (Cu(CH_3_COO)_2_·H_2_O, 98%, Merck), sodium hydroxide (NaOH, 98%, Merck), lecithin (>97% from soy, Carl-Roth), oleic acid (OAc, 90%, Merck), oleylamine (OAm, 70%, Merck), 1-octanol (>99%, Merck), ethanol (99.8%, Merck).

#### 4.1.1 Precursor preparation

##### 4.1.1.1 Copper acetate solution

Copper acetate monohydrate powder was dissolved in 1-octanol (to 25 mM) at 80 °C to obtain a deep blue solution and allowed to cool.

##### 4.1.1.2 Sodium hydroxide solution

Sodium hydroxide pellets were dissolved in a 1 : 1 solution of 1-octanol : ethanol (to 1 M). For batch experiments, this was diluted with the 1 : 1 1-octanol : ethanol mixture to make up five solutions of 0.9, 0.3, 0.1, 0.05, and 0.025 M molar concentrations.

##### 4.1.1.3 Ligand solutions

For the lecithin solution, soy-lecithin powder was dissolved in 1-octanol (150 mM). For the OAc/OAm solution, oleic acid and oleylamine were mixed in a 1 : 1 ratio and dissolved in a solution of 1-octanol (150 mM).

### 4.2 Flow reactor setup

The flow reactor (Fig. 2a) was constructed of syringe pumps Pico Elite OEM syringe pumps (Harvard Apparatus) connected to 750 μm FEP (fluorinated ethylene propylene) microfluidic tubing (Cole-Parmer, UK) to feed the precursors into the corresponding connectors. The connectors used were the T- and cross-connector pieces (Cole-Parmer, UK). For the reaction tubing, FEP tubing with an internal diameter of 750 μm was used. The connector pieces were fixed in position and the reaction tubing was fixed taut. The length of the reaction tubing was cut to correspond to a 5 min residence time, and was fed into a microfluidic flow cell (Avantes B.V., Netherlands) for *in situ* absorption measurements. This was connected to a spectrometer (AvaSpec-ULS2048XL-EVO, Avantes B.V., Netherlands) and a light source (AvaLight-DHc, Avantes B.V., Netherlands) for *in situ* absorption measurements.

### 4.3 Batch synthesis

The reaction synthesis proceeded by the mechanism described by Sonia *et al.*^34^ Five 1 mL colloidal solutions of CuO were made up by adding copper acetate and 0.9, 0.3, 0.1, 0.05, and 0.025 M of sodium hydroxide, respectively, to a vial in a 1 : 1 volume ratio. The conditions are specified in Table S1.† This approximately corresponds to the following molar concentration ratios of 1 : 1, 1 : 2, 1 : 4, 1 : 12, and 1 : 36. The mixture was stirred at room temperature until there was no longer an observable color change in the solution.

#### 4.3.1 Residence time determination

To determine an appropriate residence time of reaction in flow, we performed baseline experiments in batch to determine the required time of synthesis. The reaction was studied *via in situ* absorption spectroscopy. Reaction solutions were pipetted into a cuvette inside a cuvette holder (CUV-ALL-UV/vis, Avantes B.V, Netherlands) with a 10 mm path length, connected to a spectrometer (AvaSpec-ULS2048XL-EVO, Avantes B.V., Netherlands) and a light source (AvaLight-DHc, Avantes B.V., Netherlands). The cuvette holder was placed on a magnetic stirrer. The desired amount of copper acetate was loaded into the cuvette and stirred. After 5 s, the corresponding amount of sodium hydroxide was injected into the stirring solution and the reaction was allowed to proceed for 5 min.

### 4.4 Flow synthesis

#### 4.4.1 Single-phase flow synthesis

For flow synthesis, each precursor solution and the 1-octanol solution were loaded into separate glass syringes and placed onto Pico Elite OEM syringe pumps (Harvard Apparatus, USA). A 1 M solution of NaOH was used. The syringe pumps were then run using the conditions specified in Table S2†*via* a custom Python program.

#### 4.4.2 Single-phase ligand-guided flow synthesis

Each precursor solution was loaded into separate glass syringes, and the ligand solution and 1-octanol solutions were loaded into additional glass syringes. A 2 M solution of NaOH was used. All solutions were placed onto syringe pumps as in Fig. 2a. The syringe pumps were then run using the conditions specified *via* Table S3† a custom Python program.

### 4.5 Purification

After synthesis, subsequent NP colloidal solutions were washed with 4 mL of ethanol and 10 mL of distilled water and centrifuged at 3000 rcf for 15 min. The supernatant was then separated, and the precipitate was washed once again with 15 mL of distilled water and centrifuged at 3000 rcf for 10 min. Centrifugation was performed using a 5702 EU-IVD (Eppendorf, Germany). The collected pellet was dispersed in 5 mL of 1-octanol.

### 4.6 Characterization of CuO NPs

Characterization of CuO NP made in batch and flow was made by *in situ* and *ex situ* UV-vis absorption spectroscopy, powder X-ray diffraction (PXRD), Scanning Electron Microscopy (SEM), Fourier Transform Infrared Spectroscopy (FT-IR) and Dynamic Light Scattering (DLS).

#### 4.6.1 *In situ* UV-vis absorption

*In situ* absorption spectroscopy was conducted using an AvaSpec-ULS2048XL-EVO fibre optic spectrophotometer (Avantes B.V., Netherlands) integrated directly into the flow reactor directly post-reaction using a custom 3D printed micro flow cell with 1.5 mm optical path length, measuring from 325–800 nm wavelength.

#### 4.6.2 *Ex situ* absorbance spectroscopy

*Ex situ* absorbance spectroscopy was conducted using a UV-1800 UV/Visible Scanning Spectrophotometer (Shimadzu, Japan) measuring from 300–800 nm wavelength.

#### 4.6.3 PXRD

NP composition and grain size analysis was performed using a D8 Advance X-ray diffractometer (Bruker, USA).

#### 4.6.4 SEM/EDS

NPs were characterized using a Supra 55 VP Scanning Electron Microscope (Zeiss, Germany). A small amount of the synthesized CuO NP colloidal solution was washed, redispersed in ethanol, and drop cast onto a carbon substrate and placed onto an aluminum stub using carbon tape. For the batch sample, the NP colloidal solution was pipetted onto a silicon substrate prior to SEM. SEM size distribution analysis was performed using ImageJ analysis software from 100 individual particles from Fig. S6 and S12.†

#### 4.6.5 FT-IR

NPs were characterized using an IRXross with QATR-10 attachment (Shimadzu, Japan). A small amount of the synthesized NPs was purified and dried under vacuum to obtain the NPs in the powder form. A small quantity of the powder was subsequently analyzed. Soy-lecithin sample was analyzed directly without any modification or purification.

#### 4.6.6 DLS

DLS measurements were conducted to determine the hydrodynamic size distribution of the CuO NPs in solution. Analysis was conducted using a Zetasizer Nano (Malvern Panalytical, United Kingdom).

### 4.7 Colloidal stability

For colloidal stability measurements, UV-vis absorbance measurements (UV-1800 UV/Visible Scanning Spectrophotometer, Shimadzu, Japan) were taken every 5 days for 30 days. Additionally, DLS of the desired CuO NPs were taken fresh and after 32 days.

## Data availability

Data for this article, including all raw data and images for results presented in this article, are available at https://doi.org/10.25377/sussex.27049630.

## Author contributions

NM: conceptualization, data curation, formal analysis, investigation, methodology, visualization, writing – original draft. ZA: investigation, methodology, formal analysis. SD: formal analysis, supervision, writing – review & editing. PDH: funding acquisition, conceptualization, methodology, project administration, supervision, writing – review & editing.

## Conflicts of interest

The authors have no conflicts to declare.

## Supplementary Material

NA-OLF-D4NA00839A-s001

## References

[cit1] Zaza L., Rossi K., Buonsanti R. (2022). ACS Energy Lett..

[cit2] Santana B. D. M., Armentano G. M., Ferreira D. A. S., De Freitas C. S., Carneiro-Ramos M. S., Seabra A. B., Christodoulides M. (2024). ACS Appl. Mater. Interfaces.

[cit3] Li Y., Liu J., Chen Y., Weichselbaum R. R., Lin W. (2024). Adv. Sci..

[cit4] Gawande M. B., Goswami A., Felpin F.-X., Asefa T., Huang X., Silva R., Zou X., Zboril R., Varma R. S. (2016). Chem. Rev..

[cit5] Rehman S., Mumtaz A., Hasanain S. K. (2011). J. Nanopart. Res..

[cit6] Siddiqui H., Parra M. R., Pandey P., Qureshi M. S., Haque F. Z. (2020). J. Sci.: Adv. Mater. Devices.

[cit7] Zhu J., Li D., Chen H., Yang X., Lu L., Wang X. (2004). Mater. Lett..

[cit8] Silva N., Ramírez S., Díaz I., Garcia A., Hassan N. (2019). Materials.

[cit9] Mourdikoudis S., Menelaou M., Fiuza-Maneiro N., Zheng G., Wei S., Pérez-Juste J., Polavarapu L., Sofer Z. (2022). Nanoscale Horiz..

[cit10] Krieg F., Ong Q. K., Burian M., Rainò G., Naumenko D., Amenitsch H., Süess A., Grotevent M. J., Krumeich F., Bodnarchuk M. I., Shorubalko I., Stellacci F., Kovalenko M. V. (2019). J. Am. Chem. Soc..

[cit11] Mir W. J., Alamoudi A., Yin J., Yorov K. E., Maity P., Naphade R., Shao B., Wang J., Lintangpradipto M. N., Nematulloev S., Emwas A.-H., Genovese A., Mohammed O. F., Bakr O. M. (2022). J. Am. Chem. Soc..

[cit12] Laybourn A., Robertson K., Slater A. G. (2023). J. Am. Chem. Soc..

[cit13] Besenhard M. O., Storozhuk L., LaGrow A. P., Panariello L., Maney A., Pal S., Kiefer C., Mertz D., Tung L. D., Lees M. R., Thanh N. T. K., Gavriilidis A. (2023). Chem. Eng. J..

[cit14] El-Kadi J., Fenoaltea Pieche E., Ko S. W., Torrente-Murciano L. (2024). React. Chem. Eng..

[cit15] Pinho B., Torrente-Murciano L. (2021). Adv. Energy Mater..

[cit16] Rizk M. M., Davies G.-L. (2024). Matter.

[cit17] Gande V. V., Pushpavanam S. (2021). J. Flow Chem..

[cit18] Nikam A. V., Dadwal A. H. (2019). Adv. Powder Technol..

[cit19] Al-Antaki A. H. M., Luo X., Duan X., Lamb R. N., Hutchison W. D., Lawrance W., Raston C. L. (2019). ACS Omega.

[cit20] Tao H., Wu T., Kheiri S., Aldeghi M., Aspuru-Guzik A., Kumacheva E. (2021). Adv. Funct. Mater..

[cit21] Li S., Meng Y., Guo Y., Liu T., Stavrakis S., Howes P. D., deMello A. J. (2021). J. Mater. Chem. C.

[cit22] Volk A. A., Epps R. W., Yonemoto D. T., Masters B. S., Castellano F. N., Reyes K. G., Abolhasani M. (2023). Nat. Commun..

[cit23] Arshad Z., Blacker A. J., Chamberlain T. W., Kapur N., Clayton A. D., Bourne R. A. (2024). Curr. Opin. Green Sustainable Chem..

[cit24] Lignos I., Utzat H., Bawendi M. G., Jensen K. F. (2020). Lab Chip.

[cit25] Munyebvu N., Nette J., Stavrakis S., Howes P. D., deMello A. J. (2023). Chimia.

[cit26] Pinho B., Zhang K., Hoye R. L. Z., Torrente-Murciano L. (2022). Adv. Opt. Mater..

[cit27] Munyebvu N., Lane E., Grisan E., Howes P. D. (2022). Mater. Adv..

[cit28] Tom G., Schmid S. P., Baird S. G., Cao Y., Darvish K., Hao H., Lo S., Pablo-García S., Rajaonson E. M., Skreta M., Yoshikawa N., Corapi S., Akkoc G. D., Strieth-Kalthoff F., Seifrid M., Aspuru-Guzik A. (2024). Self-Driving Laboratories for Chemistry and Materials Science. Chemistry.

[cit29] Mándity I. M., Ötvös S. B., Fülöp F. (2015). ChemistryOpen.

[cit30] Besenhard M. O., Pal S., Gkogkos G., Gavriilidis A. (2023). React. Chem. Eng..

[cit31] Yang Z., Xu J., Zhang W., Liu A., Tang S. (2007). J. Solid State Chem..

[cit32] Singh D. P., Ojha A. K., Srivastava O. N. (2009). J. Phys. Chem. C.

[cit33] Sahooli M., Sabbaghi S., Saboori R. (2012). Mater. Lett..

[cit34] Sonia S., Jayram N. D., Suresh Kumar P., Mangalaraj D., Ponpandian N., Viswanathan C. (2014). Superlattices Microstruct..

[cit35] Usha V., Kalyanaraman S., Thangavel R., Vettumperumal R. (2015). Superlattices Microstruct..

[cit36] Bhosale M. A., Karekar S. C., Bhanage B. M. (2016). ChemistrySelect.

[cit37] Mayerhöfer T. G., Pahlow S., Popp J. (2020). ChemPhysChem.

[cit38] Li Y., Li Y., Huang L., Bin Q., Zhenyu L., Yang H., Cai Z., Chen G. (2013). J. Mater. Chem. B.

[cit39] Demel J., Zhigunov A., Jirka I., Klementová M., Lang K. (2015). J. Colloid Interface Sci..

[cit40] Ganga B. G., Santhosh P. N. (2014). J. Alloys Compd..

[cit41] Sagadevan S., Pal K., Chowdhury Z. Z. (2017). J. Mater. Sci.: Mater. Electron..

[cit42] Dagher S., Haik Y., Ayesh A. I., Tit N. (2014). J. Lumin..

[cit43] Salazar-Alvarez G., Muhammed M., Zagorodni A. A. (2006). Chem. Eng. Sci..

[cit44] Zhang K., Gao Y., Pinho B., Hoye R. L. Z., Stranks S. D., Torrente-Murciano L. (2023). Chem. Eng. J..

[cit45] Huang W., Blinov N., Kovalenko A. (2015). J. Phys. Chem. B.

[cit46] Mohsen-Nia M., Amiri H., Jazi B. (2010). J. Solution Chem..

[cit47] Aissa M. A. B., Tremblay B., Andrieux-Ledier A., Maisonhaute E., Raouafi N., Courty A. (2015). Nanoscale.

[cit48] Akkerman Q. A., Nguyen T. P. T., Boehme S. C., Montanarella F., Dirin D. N., Wechsler P., Beiglböck F., Rainò G., Erni R., Katan C., Even J., Kovalenko M. V. (2022). Science.

[cit49] Lazzari S., Theiler P. M., Shen Y., Coley C. W., Stemmer A., Jensen K. F. (2018). Langmuir.

[cit50] Li W., Taylor M. G., Bayerl D., Mozaffari S., Dixit M., Ivanov S., Seifert S., Lee B., Shanaiah N., Lu Y., Kovarik L., Mpourmpakis G., Karim A. M. (2021). Nanoscale.

[cit51] Ortiz N., Skrabalak S. E. (2012). Angew. Chem., Int. Ed..

[cit52] Zhitomirsky D., Kramer I. J., Labelle A. J., Fischer A., Debnath R., Pan J., Bakr O. M., Sargent E. H. (2012). Nano Lett..

[cit53] Sudha V., Murugadoss G., Thangamuthu R. (2021). Sci. Rep..

[cit54] Kuligowski J., Quintás G., Esteve-Turrillas F. A., Garrigues S., de la Guardia M. (2008). J. Chromatogr. A.

[cit55] Besenhard M. O., Pal S., Storozhuk L., Dawes S., Thanh N. T. K., Norfolk L., Staniland S., Gavriilidis A. (2023). Lab Chip.

