# Supplementary material for: Flow synthesis and multidimensional parameter screening enables exploration and optimization of copper oxide nanoparticle synthesis
Source: Nanoscale Adv. 2024 Dec 3;7(2):495–505. doi: 10.1039/d4na00839a (PMC11613990; doi:10.1039/d4na00839a)
Supplement: NA-007-D4NA00839A-s001 [file NA-007-D4NA00839A-s001.pdf]

Supplementary Information (SI) for Nanoscale Advances.
This journal is © The Royal Society of Chemistry 2024

Flow synthesis and multidimensional parameter screening allows exploration and optimization of copper oxide nanoparticle synthesis: Supplementary Information

1. RESIDENCE TIME DETERMINATION

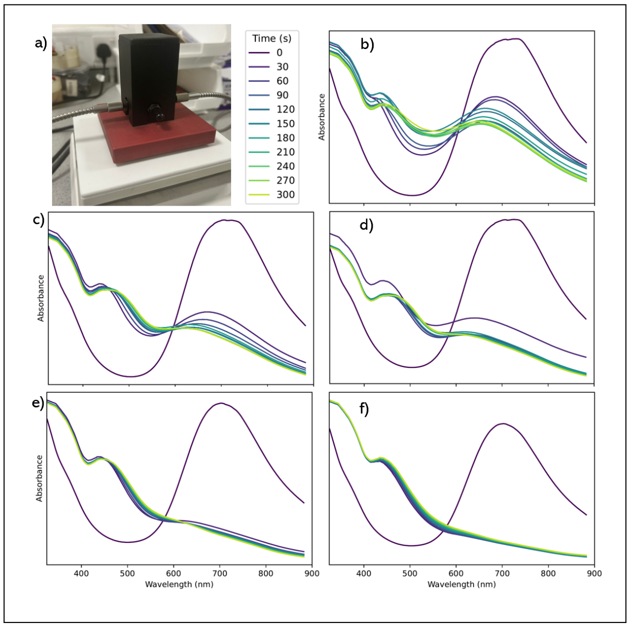

Fig. S1. (a) Apparatus for experiments to monitor the UV-vis absorbance spectra evolution of the transition of copper acetate following the addition of various molar ratios of NaOH/Cu^{2+}: b) 1, c) 2, d) 4, e)12 and f) 36

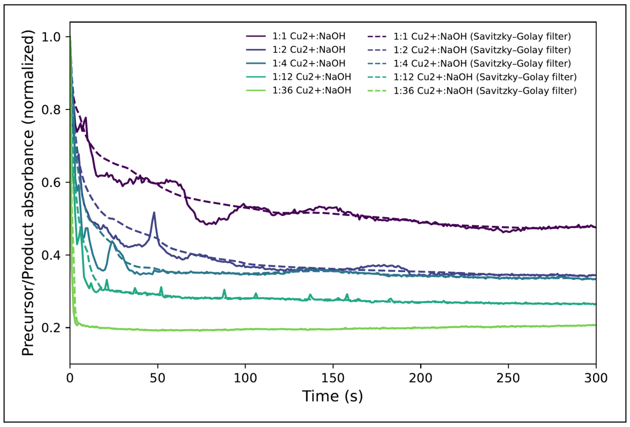

Fig. S2. Ratio of the intensity of product peak against the precursor peak over 300 seconds post-injection of NaOH. A Savitsky-Golay filter (dashed lines) was applied to smooth the data and clearly interpret the rate of change.

2. BATCH SYNTHESIS AND CHARACTERIZATION

Precursor UV-vis absorbance data

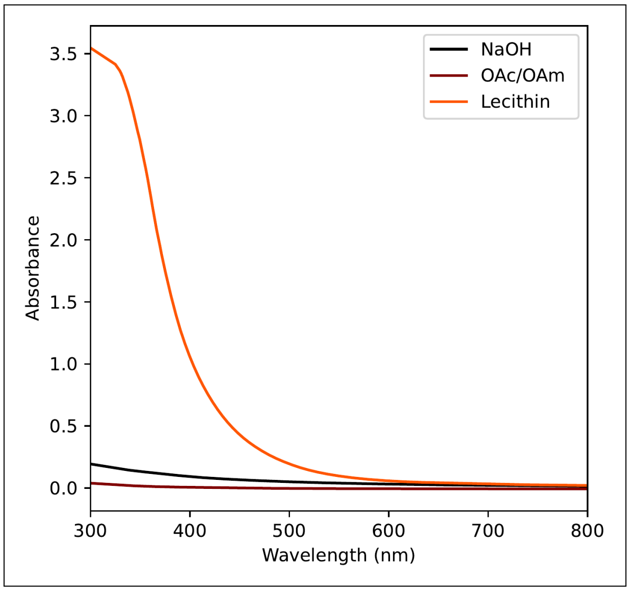

Fig. S3. UV-vis absorbance spectra for 0.5M NaOH, 0.15M lecithin and 0.15M oleic acid/oleylamine precursors in 1-octanol

Synthesis parameters

Table S1. Corresponding precursor volumes for CuO batch experiments

$Cu(OAc)_2$ (uL)	NaOH (uL)	Total Volume (uL)	$Cu(OAc)_2$ conc. (mM)	NaOH conc. (mM)
500	500	1000	12.5	12.5
500	500	1000	12.5	25
500	500	1000	12.5	50
500	500	1000	12.5	150
500	500	1000	12.5	450

NB: Concentrations are the relative concentration of precursors after mixing.

Characterization

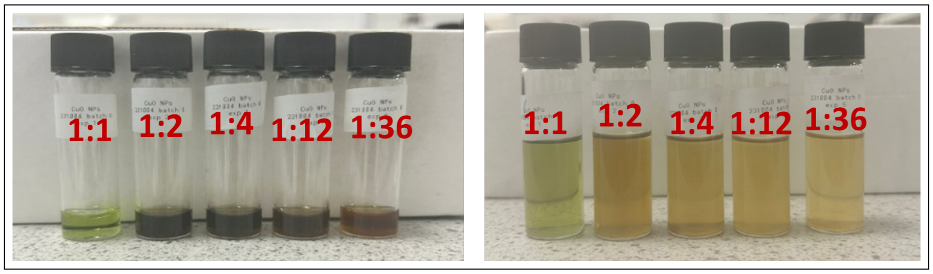

Fig. S4. Photograph of the as-prepared solutions after 5 minute reaction (pre-purification), synthesized under flask conditions pre- (L) and post- (R) dilution with 1-octanol.

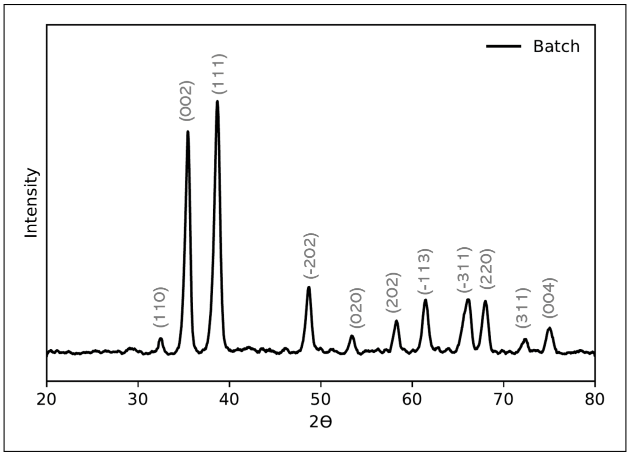

Fig. S5. XRD spectra for the CuO nanoparticles synthesised using a 1:12 Cu2+: NaOH ratio under batch conditions.

The Debye Scherrer equation can be used to estimate the crystallite grain size from the XRD spectra [1]:

$$D = \frac{K\lambda}{\beta \cos\theta} \tag{S1}$$

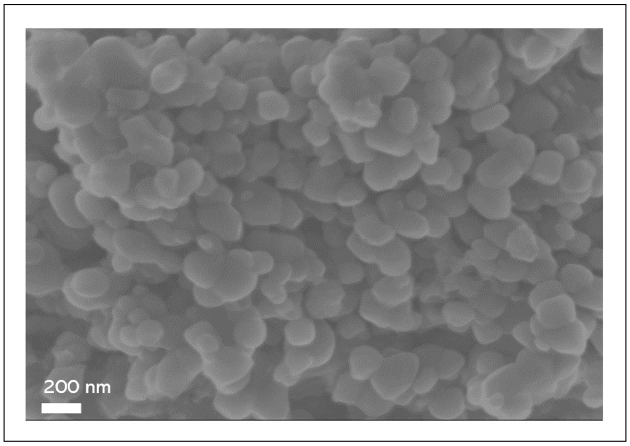

Fig. S6. SEM image for the CuO nanoparticles synthesised using a 1:12 Cu2+:NaOH ratio under batch conditions.

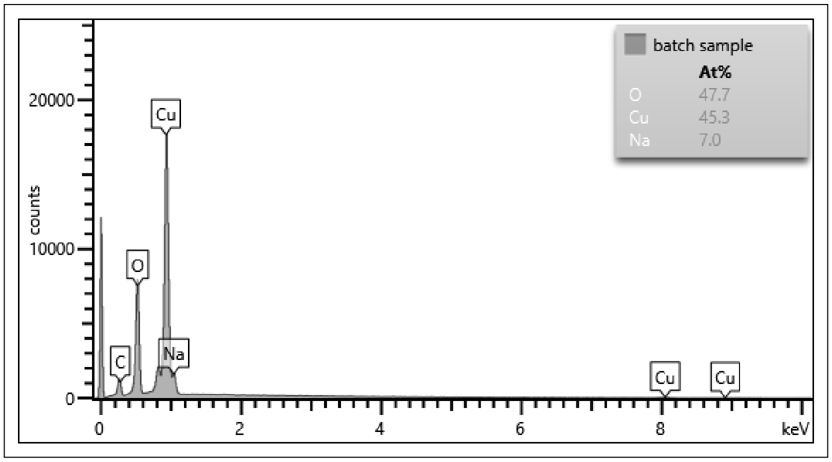

Fig. S7. EDS Spectra for the CuO nanoparticles synthesised using a 1:12 Cu2+:NaOH ratio under batch conditions.

3. FLOW SYNTHESIS AND CHARACTERIZATION

Synthesis parameters

Table S2. Corresponding flow rates for CuO flow experiments

$Cu(OAc)_2$ (uL/min)	NaOH (uL/min)	1-Octanol (uL/min)	$Cu(OAc)_2$ conc. (mM)	NaOH conc. (mM)
50	1.25	48.75	12.5	12.5
50	2.5	47.5	12.5	25
50	5	45	12.5	50
50	15	35	12.5	150
50	45	5	12.5	450

NB: Concentrations are the relative concentration of precursors after mixing.

Characterization

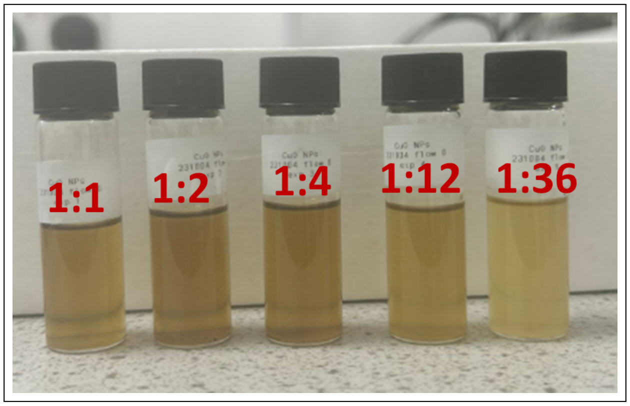

Fig. S8. Photograph of the as-prepared solutions after 5 minute reaction (pre-purification), synthesized under flow conditions and post-dilution with 1-octanol.

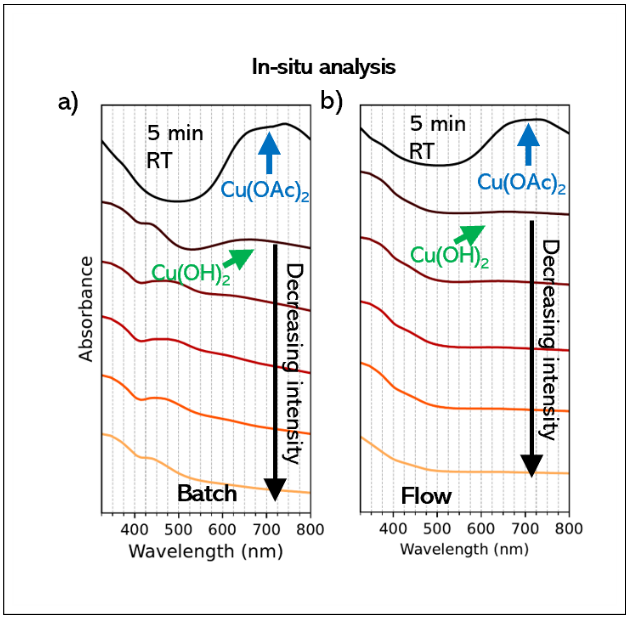

Fig. S9. Final UV-vis absorption spectrum for the solutions after 5 min reaction time (RT) upon the addition of various molar ratios of NaOH for a) batch and b)flow conditions

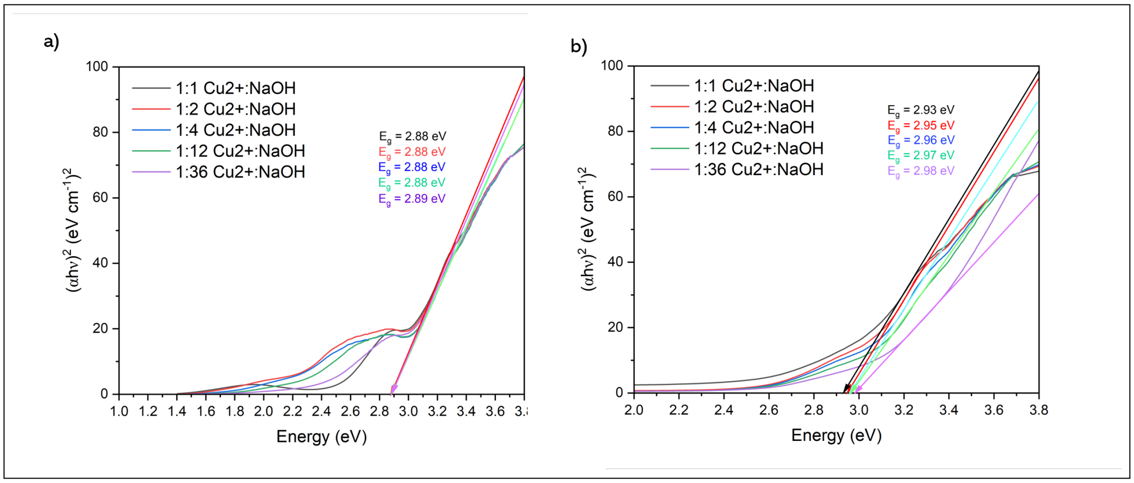

Fig. S10. Tauc plots and estimated band gaps for various Cu^{2+}:NaOH ratios for CuO NPs synthesized under a) batch and b) flow conditions

The Tauc equation can be used to estimate the band gap energy from the UV-vis spectroscopy data:

$$(\alpha h\nu)^{1/n} = A(h\nu - E_g) \quad \text{(S2)}$$

where α is absorption coefficient being a function of wavelength $\alpha(\lambda)$, h is Planck constant, E_g is an optical band gap of a semiconductor, ν is frequency, A is proportionality constant, and n is Tauc coefficient. This study investigated the direct (allowed) transitions, where n= 1/2.[2]

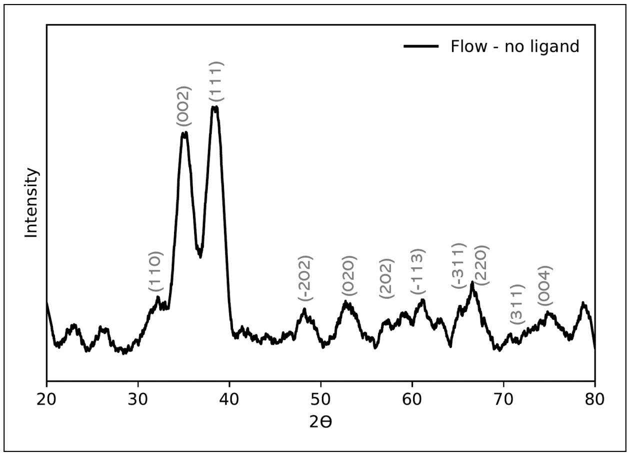

Fig. S11. XRD pattern for the CuO nanoparticles synthesised using a 1:12 Cu2+: NaOH ratio under flow conditions.

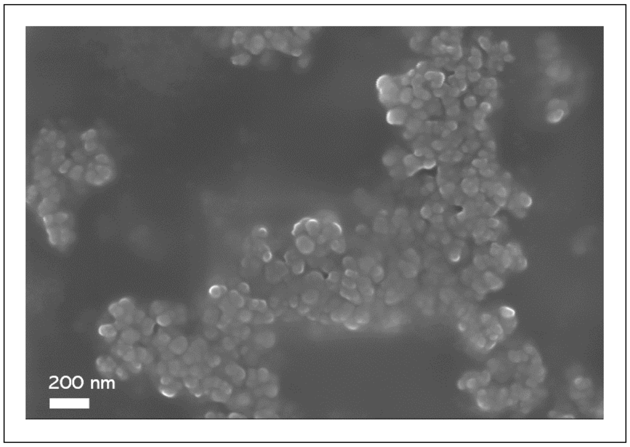

Fig. S12. SEM image for the CuO nanoparticles synthesised using a 1:12 Cu2+:NaOH ratio under flow conditions.

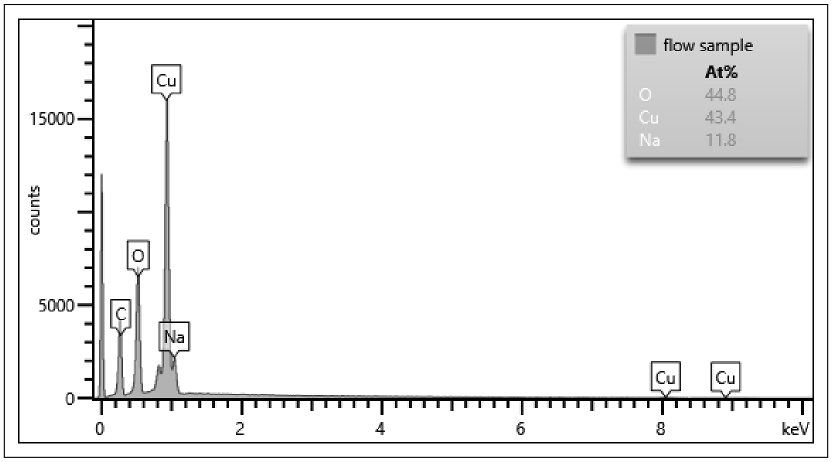

Fig. S13. EDS Spectra for the CuO nanoparticles synthesised using a 1:12 Cu2+:NaOH ratio under flow conditions.

4. MAPPING THE BAND GAP RESPONSE TO VARYING LECITHIN AND PRECURSOR CONCENTRATIONS

Parameters

Table S3. Corresponding flow rates for the parameter scan experiments

$Cu(OAc)_2$ (uL/min)	NaOH (uL/min)	Ligand (uL/min)	Octanol (uL/min)	NaOH/Cu^{2+}	Ligand conc. (mM)
50	3.125	0	46.875	5	0
50	3.125	0.833	46.042	5	1.25
50	3.125	4.167	42.708	5	6.25
50	3.125	8.33	38.545	5	12.5
50	3.125	12.5	34.375	5	18.75
50	7.8125	0	42.1875	12.5	0
50	7.8125	0.833	41.3545	12.5	1.25
50	7.8125	4.167	38.0205	12.5	6.25
50	7.8125	8.33	33.8575	12.5	12.5
50	7.8125	12.5	29.6875	12.5	18.75
50	12.5	0	37.5	20	0
50	12.5	0.833	36.667	20	1.25
50	12.5	4.167	33.333	20	6.25
50	12.5	8.33	29.17	20	12.5
50	12.5	12.5	25	20	18.75
50	17.1875	0	32.8125	27.5	0
50	17.1875	0.833	31.9795	27.5	1.2495
50	17.1875	4.167	28.6455	27.5	6.2505
50	17.1875	8.33	24.4825	27.5	12.495
50	17.1875	12.5	20.3125	27.5	18.75
50	21.875	0	28.125	35	0
50	21.875	0.833	27.292	35	1.2495
50	21.875	4.167	23.958	35	6.2505
50	21.875	8.33	19.795	35	12.495
50	21.875	12.5	15.625	35	18.75

NB: Concentrations are the relative concentration of precursors after mixing.

Characterization

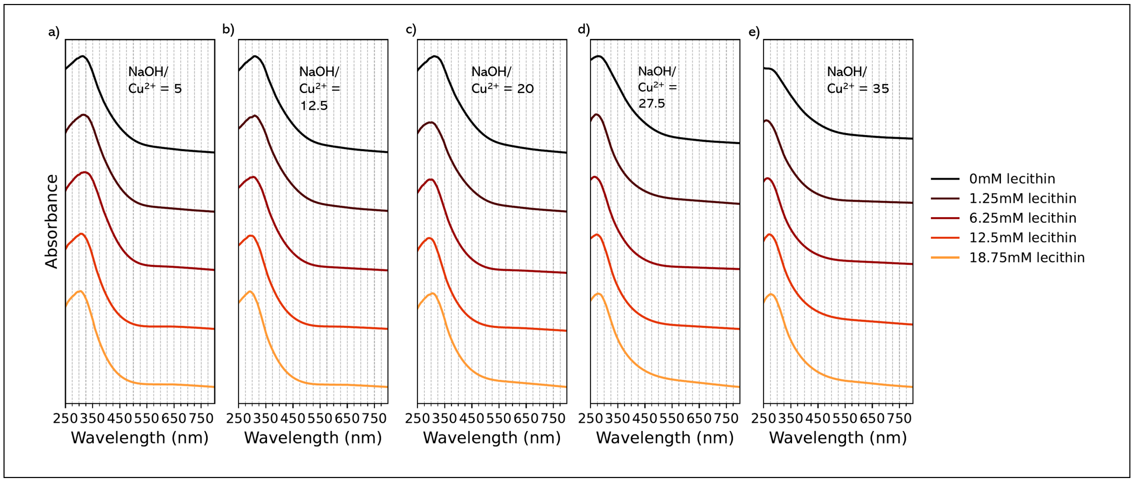

Fig. S14. UV-vis absorbance spectra for various concentrations of lecithin for NaOH/Cu^{2+} ratios of a) 5, b) 12.5, c) 20, d) 27.5 and e) 35

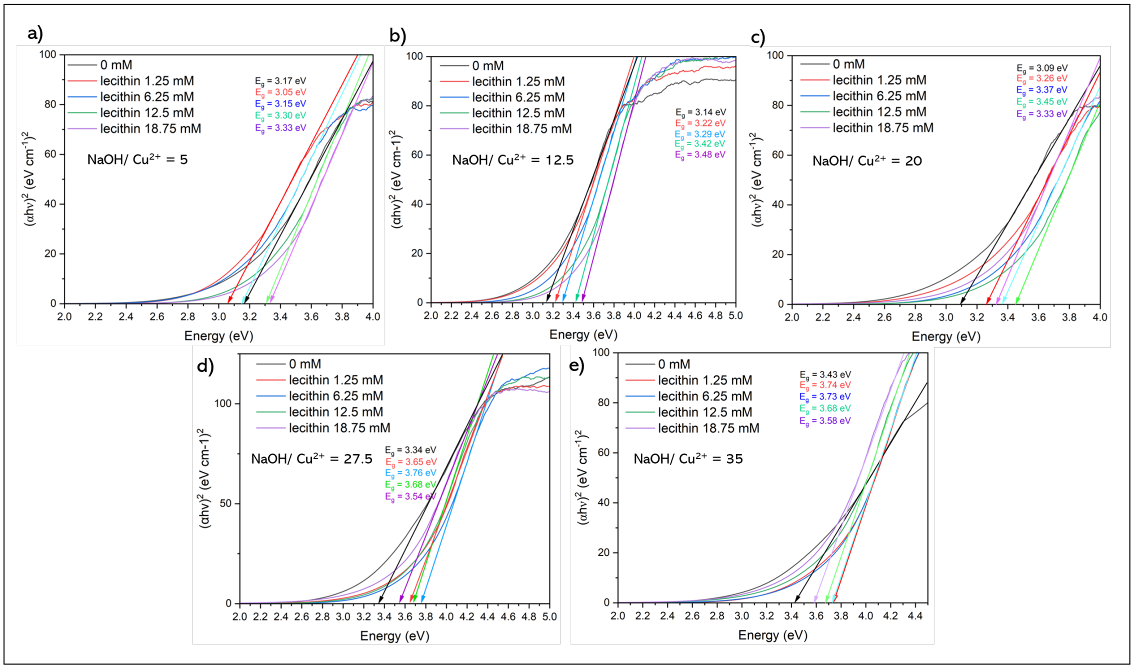

Fig. S15. Tauc plots and estimated band gaps for various concentrations of lecithin for NaOH/Cu^{2+} ratios of a) 5, b) 12.5, c) 20, d) 27.5, and e) 35

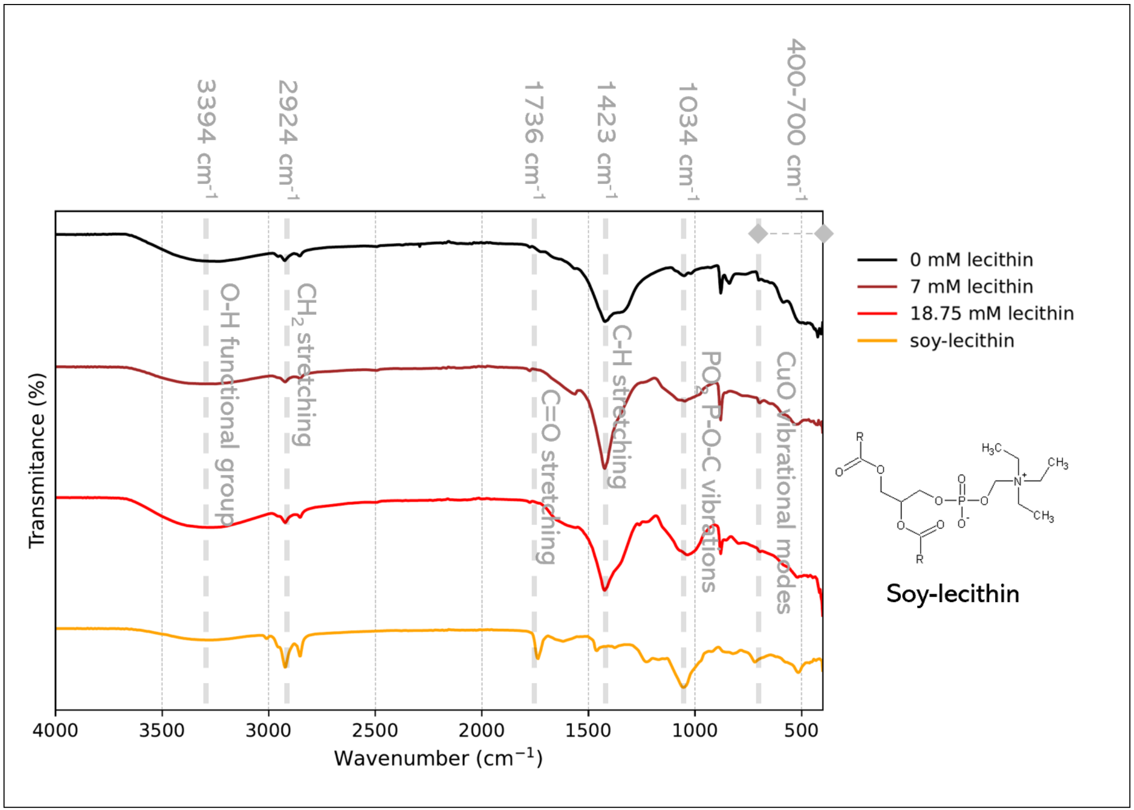

Fig. S16. FT-IR spectra for various concentrations of lecithin synthesized under flow conditions

Stability Measurements

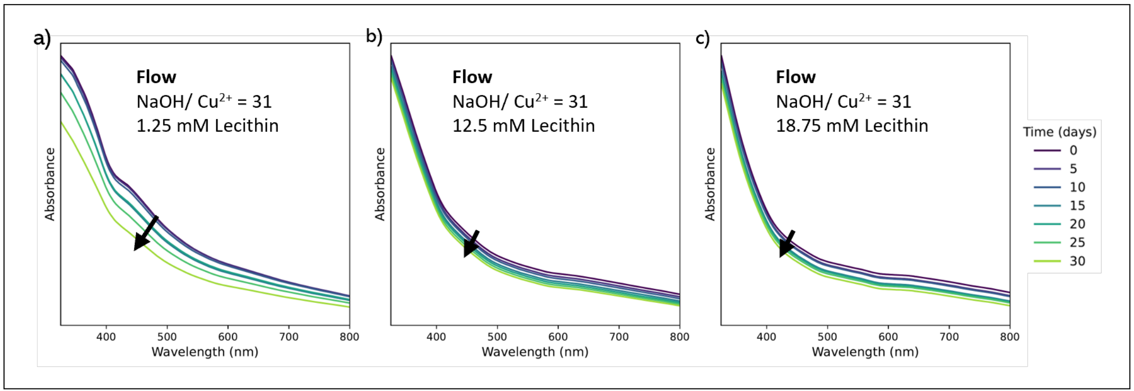

Fig. S17. Evolution of UV-Vis absorption spectra of the colloidal suspension of samples of CuO synthesized in flow recorded over 30 days for lecithin concentrations of a) 1.25 mM, b) 6.25 mM, and c) 18.75 mM.

REFERENCES

1. A. O. Bokuniaeva and A. S. Vorokh, "Estimation of particle size using the Debye equation and the Scherrer formula for polyphasic TiO_2 powder," J. Phys.: Conf. Ser. **1410**, 012057 (2019).
2. Haryński, A. Olejnik, K. Grochowska, and K. Siuzdak, "A facile method for Tauc exponent and corresponding electronic transitions determination in semiconductors directly from UV–Vis spectroscopy data," Opt. Mater. **127**, 112205 (2022).
